# Sclerostin and Osteocalcin: Candidate Bone-Produced Hormones

**DOI:** 10.3389/fendo.2021.584147

**Published:** 2021-03-10

**Authors:** Jialiang S. Wang, Courtney M. Mazur, Marc N. Wein

**Affiliations:** ^1^ Endocrine Unit, Department of Medicine, Massachusetts General Hospital, Harvard Medical School, Boston, MA, United States; ^2^ Broad Institute of Massachusetts Institute of Technology (MIT) and Harvard, Cambridge, MA, United States; ^3^ Harvard Stem Cell Institute, Cambridge, MA, United States

**Keywords:** osteoblast, osteocyte, osteocalcin, sclerostin, bone homeostasis

## Abstract

In addition to its structural role, the skeleton serves as an endocrine organ that controls mineral metabolism and energy homeostasis. Three major cell types in bone - osteoblasts, osteoclasts, and osteocytes – dynamically form and maintain bone and secrete factors with systemic activity. Osteocalcin, an osteoblast-derived factor initially described as a matrix protein that regulates bone mineralization, has been suggested to be an osteoblast-derived endocrine hormone that regulates multiple target organs including pancreas, liver, muscle, adipose, testes, and the central and peripheral nervous system. Sclerostin is predominantly produced by osteocytes, and is best known as a paracrine-acting regulator of WNT signaling and activity of osteoblasts and osteoclasts on bone surfaces. In addition to this important paracrine role for sclerostin within bone, sclerostin protein has been noted to act at a distance to regulate adipocytes, energy homeostasis, and mineral metabolism in the kidney. In this article, we aim to bring together evidence supporting an endocrine function for sclerostin and osteocalcin, and discuss recent controversies regarding the proposed role of osteocalcin outside of bone. We summarize the current state of knowledge on animal models and human physiology related to the multiple functions of these bone-derived factors. Finally, we highlight areas in which future research is expected to yield additional insights into the biology of osteocalcin and sclerostin.

## Introduction

Traditionally considered as a structural organ, the skeleton provides mechanical support and protection for soft organs and facilitates mobility. To maintain skeletal integrity, the three major cell types within bone – osteoblasts, osteocytes, and osteoclasts – remodel bone through coupled processes. Osteoclasts are multinucleated hematopoietic cells of the monocyte-macrophage lineage that resorb bone along its surfaces (1). Osteoblasts originate from mesenchymal progenitor cells and produce bone matrix proteins to facilitate bone formation on the surface (2, 3). Some osteoblasts acquire long dendritic processes and embed within bone to become terminally-differentiated osteocytes (4). Osteocytes remodel their surrounding bone matrix and orchestrate the activity of osteoblasts and osteoclasts (4).

One way that osteoblasts, osteocytes, and osteoclasts communicate is through production of paracrine signaling molecules that act on neighboring cells. Well known bone-derived paracrine factors include sclerostin (osteocyte-derived inhibitor of Wnt signaling), receptor activator of NF-кB ligand (RANKL, a key regulator of osteoclast differentiation produced mainly by mesenchymal osteoblast-lineage cells), monocyte/macrophage colony stimulating factor (M-CSF, osteoblast-derived stimulator of myeloid cell survival and osteoclastogenesis) and osteoprotegerin (OPG, osteoblast-derived inhibitor of osteoclastogenesis) (5–10). Beyond well-established bone-derived paracrine-acting factors that participate in cross-talk between cell types within bone, bone-derived ***endocrine*** factors have also been reported.

Other than its classic structural role, the paracrine and endocrine functions of bone are essential for organismal homeostasis. Fibroblast growth factor 23 (FGF-23) is mainly secreted by osteoblasts and osteocytes and plays an important role in regulating phosphate homeostasis (11). Osteocalcin (OCN), the most abundant non-collagenous bone matrix protein, is produced specifically by osteoblasts and is suggested to regulate the biological processes of multiple organs including bone, brain, liver, pancreas, testes, muscle, the parasympathetic nervous system, and adipose tissue (11). Recent studies demonstrate that osteoblast-derived lipocalin 2 (LCN2) regulates glucose tolerance, insulin sensitivity, and insulin secretion to maintain glucose homeostasis (12). In addition to its paracrine roles, effects of sclerostin on adipose tissue and mineral metabolism have also been reported recently, with an additional possible role in preventing vascular calcification (13–15). Given the established role of FGF-23 in regulating renal phosphate handling and the relatively limited information on LCN2, this review will focus on osteocalcin and sclerostin. Here, we will bring together the recent data on osteocalcin and sclerostin in bone and distant target organs, primarily focusing on important new insights into the role of these circulating factors learned from animal models.

## Osteocalcin

Osteocalcin, or bone γ-carboxyglutamic acid (Gla) protein, is an osteoblast-derived circulating protein. Osteocalcin is initially synthesized as a prohormone (95 amino acids) and then cleaved to form the mature peptide (46 amino acids) that contains three γ-carboxyglutamic acid residues at positions 13, 17 and 20 (16). In human, the mature peptide has 49 amino acids and is γ-carboxylated at positions 17, 21 and 24 (17). γ-carboxylation increases the affinity of osteocalcin to the mineral component of the extracellular matrix. This leads to the accumulation of γ-carboxylated osteocalcin in bone (18). Osteoclast resorption, on the other hand, creates an acidic environment where osteocalcin is de-carboxylated. Under-carboxylated osteocalcin has lower affinity to bone matrix and is released into the bloodstream where it can function as an endocrine hormone (19, 20).

OCN is encoded by a single gene (*BGLAP*) in human, while mice have a cluster of three genes (*Bglap*, *Bglap2*, and *Bglap3*) within a 25 kb genomic region (21–23). *Bglap* and *Bglap2* are highly expressed in bone, while *Bglap3* has a relatively lower expression in bone, but higher expression in kidney and lung (23–25). The amino acid sequences of γ-carboxylated Ocn encoded by *Bglap* and *Bglap2* are identical. The sequence encoded by *Bglap3* is different from the sequences encoded by *Bglap* and *Bglap2* by four amino acid residues (26).

## Osteocalcin in Skeletal Development

To study and understand the role of osteocalcin in bone formation, Karsenty and colleagues generated the first *Ocn-*deficient mouse (*Osc^-^/Osc^-^*) in 1996 (27). Mice homozygous for the deletion of *Bglap* and *Bglap2* were generated in embryonic stem (ES) cells by homologous recombination. Exon4 of *Bglap* and the entire sequence of *Bglap2* were replaced by a PGK-Neo cassette. *Osc^-^/Osc^-^* pups are viable, fertile and there are no skeletal defects at birth. At 6 and 9-months of age, osteocalcin null mice showed significantly increased bone mass, bone strength and bone formation, without changes in osteoblast numbers or bone resorption. A subsequent study on the role of osteocalcin in extracellular matrix using fourier-transform infrared imaging (FTIR) showed larger hydroxyapatite crystal size (28). More recently, it was reported that osteocalcin null mice on a pure C57BL/6J background show increased carbonate-to-mineral ratio in cortical bone (29).

More recently, studies of two independently-generated osteocalcin knockout mouse models were published in *PLOS Genetics*. In an article by Diegel and colleagues, the authors generated a *Bglap* and *Bglap2* double-knockout (*Bglap/2^dko/dko^*) strain using CRISPR/Cas9-mediated gene editing (26). More specifically, they designed guide RNAs that target *Bglap* and *Bglap2*, but not *Bglap3.* In one founder allele, a 6.8-kb fragment was deleted which leads to a functional junction between exon 2 of *Bglap* to exon 4 of *Bglap2*. The authors reported no differences in bone mass or bone strength between homozygous mutants and wild-type mice. Further FTIR analysis revealed that *Bglap/2^dko/dko^* mice have increased crystal size and higher carbonate-to-mineral ratio. Moriishi and colleagues also reported a novel *Ocn* knockout strain (*Ocn^-/-^*) using homologous recombination by replacing the genomic region encompassing *Bglap* and *Bglap2* with the gene conferring neomycin resistance (30). Both trabecular and cortical bone mass are similar in wild-type and *Ocn* knockout mice. While most skeletal parameters analyzed were normal, these authors did observe disrupted orientation of hydroxyapatite crystals along collagen fibrils in their *Ocn* ^-/-^ strain.

Several possible explanations exist for differences in skeletal phenotypes among three osteocalcin knockout models, including mouse genetic background, sex, differences in age of analysis, effects of mutated alleles on neighboring genes and, potentially, technical differences between homologous recombination and CRISPR/Cas9-mediated gene editing. In all 3 osteocalcin-null models, complete genome sequencing has not been performed, which leaves open the possibility that ‘off-target’ effects related to traditional or CRISPR/Cas9-mediated target gene modification may drive phenotypic differences. Though the specificity of Cas9 is determined by the 20-base pair (bp) sequence of the sgRNA and the NGG trinucleotide (the protospacer-adjacent motif, PAM) adjacent to the target sequence, off-target mutations can be induced at sites that differ slightly from the on-target sites (31–33). Sensitive and comprehensive approaches are therefore required to detect off-target sites. Currently developed and widely-adapted methods include deep sequencing, web-based in silico prediction tools, and ChIP-seq (34–37). Compared to CRISPR-Cas9 gene editing, conventional homologous recombination leads to rare off-target effects (38). Whole genome sequencing of all three osteocalcin mutant mouse strains may therefore prove useful to clarify potential differences between these models at the level of locus modification and potential off-target changes. *Osc^-^/Osc^-^* mice from Karsenty and colleagues were initially generated and characterized in the mixed BL6/129 background; more recently, studies related to energy metabolism, male fertility and neurobiology on that studies have been performed on a ‘pure’ 129 background (39–41). *Bglap/2^dko/dko^* mice generated by Diegel and colleagues were on a mixed BL6/C3H background (back-crossed to C57BL/6 for 2 generations). *Ocn^-/-^* mice were generated in the pure C57BL/6 background by Moriishi and colleagues. Studies from Berezovaska and colleagues do support genetic background as a contributing factor to different osteocalcin null phenotypes (29). When *Osc^-^/Osc^-^* mice were backcrossed to pure C57BL/6J mice (more than 8 generations), reduced bone strength was observed. Other than genetic background, Moriishi et al. used mice with different ages and sexes compared to *Osc^-^/Osc^-^* mice generated by Karsenty and colleagues.

**Figure 1:**
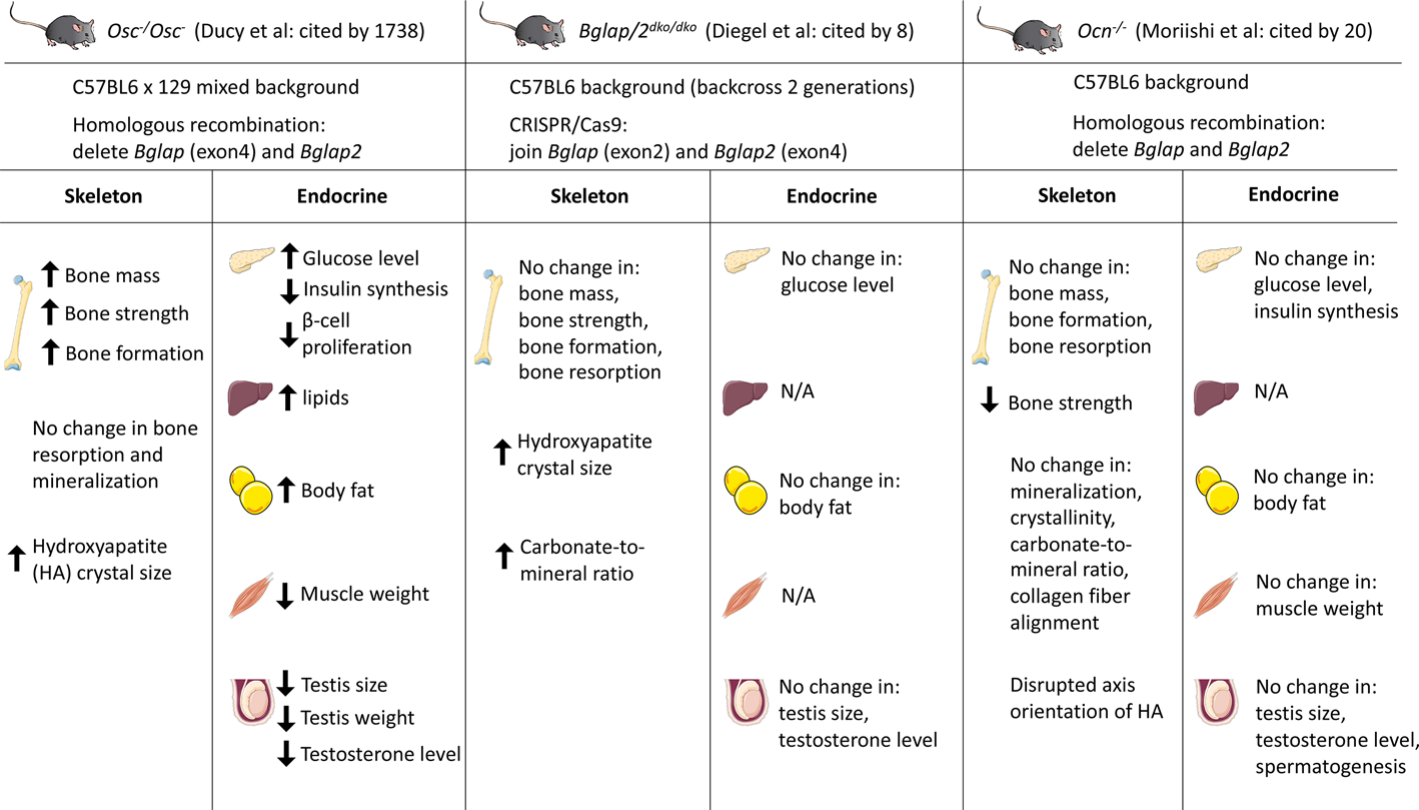
Summary of three osteocalcin null mouse models and their observed phenotypes in different tissues. Karsenty and colleagues generated the first *Ocn*-deficient mouse (*Osc^-^/Osc^-^*) in 1996. *Bglap* and *Bglap2* were deleted in embryonic stem cells using homologous recombination. Diegel and colleagues generated the *Bglap and Bglap2* double-knockout (*Bglap/2^dko/dko^*) strain using CRISPR/Cas9-mediated gene editing. Moriishi and colleagues generated the novel *Ocn* knockout mouse (*Ocn* ^-/-^) using homologous recombination by replacing the genomic region encompassing *Bglap* and *Bglap2* with the neomycin resistant gene. *Osc* ^-^/*Osc* ^-^ mice showed increased bone mass and bone formation, while *Bglap/2^dko/dko^* and *Ocn* ^-/-^ mice showed similar bone mass/formation compared to their control littermates. Many investigators reported the endocrine role of osteocalcin by studying *Osc^-^/Osc^-^ mice*. Circulating osteocalcin stimulates β-cell proliferation and insulin synthesis in pancreas. Under-carboxylated osteocalcin may also increase insulin sensitivity in adipose tissue and liver. Elevated osteocalcin increases nutrient uptake in muscle and exercise capacity. In addition, osteocalcin promotes male fertility by increasing testosterone synthesis. However, initial analyses on *Bglap/2^dko/dko^* and *Ocn^-/-^* mice demonstrated that osteocalcin has no effect on glucose level, body fat, muscle weight, and testis size. N/A, not assessed. This figure was created using Servier Medical Art templates, which are licensed under a Creative Commons Attribution 3.0 Unported License; https://smart.servier.com

In summary, there are both consistent and inconsistent findings when comparing skeletal traits in the three reported osteocalcin knockout mouse models (**Figure 1**). Beyond some questions about the role of osteocalcin in bone, different potential roles of osteocalcin as an endocrine factor will be discussed below in detail. At present, additional work is needed to clarify this active controversy (42–45). Potential future strategies that could be considered to address this controversy might include a blinded, side-by-side comparison of the 3 different osteocalcin-null models with appropriate age- and sex-matched littermate controls. This approach may help to tease apart the relative role of methodological differences in assessing metabolic phenotypes (see below) versus inherent intrinsic differences between different osteocalcin mutant strains in driving different observations. An additional, constructive approach may be to backcross to ‘purity’ all mouse models on the same genetic background and then rigorously re-analyze each model.

## Osteocalcin in Energy Metabolism (Pancreas, Liver, Adipose and Muscle)

The first demonstration of a potential endocrine function of osteocalcin came with the description that *Osc^-^/Osc* ^-^ mice have high blood glucose levels, increased fat mass, glucose intolerance due to decreased insulin synthesis and beta cell proliferation, and insulin resistance (39). In contrast, *Esp^-/-^* mice with ablation of osteo-testicular protein tyrosine phosphatase have opposite metabolic phenotypes as *Osc* ^-/-^ animals: decreased fat mass, increased β-cell proliferation and enhanced insulin sensitivity (46, 47). The authors went on to show that *Esp^-/-^* mice exhibit a gain-of-function of osteocalcin phenotype since the encoded phosphatase is a negative regulator of osteocalcin activation. *Esp*-deficient mice have decreased γ-carboxylated osteocalcin and therefore show increased serum osteocalcin levels. Further studies showed that exogenous un-carboxylated osteocalcin can also increase glucose tolerance and insulin sensitivity (39, 48, 49). Reciprocal regulation of osteocalcin by insulin has also been demonstrated: insulin-treatment of MC3T3-E1 cells increased osteocalcin promoter activity and gene expression (50). Mice with deletion of the insulin receptor from osteoblasts show increased body fat, impaired glucose metabolism, reduced levels of circulating uncarboxylated osteocalcin, and impaired insulin sensitivity (49, 50). These analyses suggest a bone-pancreas endocrine loop where insulin induces osteocalcin expression and then in turn promotes insulin secretion. The receptor Gprc6a may mediate the osteocalcin function in pancreatic islets. *In vivo*, *Gprc6a^-/-^* pancreas showed decreased β-cell mass, β-cell proliferation and impaired capacity of insulin secretion (48). However, Diegel and colleagues showed no differences in blood glucose (with or without fasting) and body weight between *Bglap/2^dko/dko^* and wild-type littermates (26). These phenotypes align with previous reports on *Bglap* knockout rats. Osteocalcin null rats exhibit no change in glucose levels and insulin resistance (51). Moriishi and colleagues also examined the role of osteocalcin in glucose metabolism and observed similar blood glucose between *Ocn^-/-^* and wild-type mice in both sexes at all ages. Both glucose tolerance tests (GTTs) and insulin tolerance tests (ITTs) showed no change of serum glucose levels in *Ocn^-/-^* mice in both sexes, at all ages and with both normal and high fat diet fed animals (30).

At present there is no unifying explanation to account for these apparently discrepant findings. A large body of evidence published in high quality, peer-reviewed journals strongly supports a role for osteocalcin in regulating energy metabolism (43, 52). The recent CRISPR/Cas9-generated osteocalcin-null models reported limited numbers of mice analyzed for analysis of glucose metabolism phenotypes. As detailed above, a ‘contemporaneous replication’ strategy (53), if possible, may be helpful to build confidence in potentially-controversial preclinical findings. In addition, in an important perspective relevant to this issue, the topic of inter-lab variability in the setting of preclinical energy metabolism models has recently been addressed in a productive manner by top scientists from the pharmaceutical industry (54). As noted above, differences in strain background and off-target effects may play an important role in driving different observations between osteocalcin mutant strains reported to date. In addition, important methodologic differences must be acknowledged in how metabolic phenotypes were assessed in the ‘newer’ osteocalcin mutant strains versus the extensive metabolic phenotyping performed by Karsenty and colleagues over many years and multiple publications. For example, Diegel and colleagues measured fasting blood glucose in n=8-11 six month old *Bglap/2^dko/dko^* mice, without measuring insulin levels, performing dynamic testing such as oral glucose tolerance tests or insulin tolerance tests, or investigating glucose-related phenotypes at other ages (26). Moriishi et al. did perform dynamic testing (30), though methodologic differences appear to exist between doses of glucose and insulin used in their studies versus those previous reported by Karsenty and colleagues (39). In addition, it is possible that differences in housing conditions (bedding, animal diet, local microbiome), sex of mice analyzed, and experimental sample size may be present between the different studies as well. These same methodologic issues may contribute to differences noted between strains with respect to assessment of fertility and gonadal hormone levels. **Figure 1** summarizes the currently-available mouse osteocalcin mutant models.

In addition to glucose intolerance and insulin resistance, *Osc^-^/Osc^-^* mice showed liver steatosis, adipose tissue inflammation, and reduced exercise capacity (46, 47). Since osteocalcin knockout mice (*Osc^-^/Osc^-^*) showed accumulation of body fat, several research groups examined the role of Ocn as a potential mediator of crosstalk between bone and adipose tissue. The direct role of osteocalcin in adipocytes was demonstrated first through *in vitro* analyses. *In vitro* uncarboxylated osteocalcin treatment of adipocytes increased the expression of peroxisome-proliferator-activated receptor γ (master regulator of adipogenesis) and adiponectin (an adipokine that regulates glucose and lipid metabolism) *via* the Gprc6a receptor (49, 55). *In vivo* treatment of mice with recombinant osteocalcin can also upregulate adipocyte adiponectin in white and brown adipose tissues (46). Though *Gprc6a^-/-^* mice also had increased fat mass, further studies are needed to reveal whether osteocalcin interacts with Gprc6a or other potential receptors in adipose tissue (56). Two studies reported that adiponectin plays a role in regulating insulin sensitivity in high fat diet-fed mice (57) and adiponectin can regulate bone mass in normal diet-fed mice without affecting glucose levels (58). It still remains unknown how osteocalcin affects fat mass. Uncarboxylated osteocalcin treatment can promote the expression of adipose triglyceride lipase (ATGL) and further lead to the induction of lipolysis though the cAMP-PKA-ERK-CREB signaling *in vitro* (59). Livers in *Osc^-^/Osc^-^* mice show accumulation of lipids and steatosis (49). Mice treated with osteocalcin have no accumulation of lipids and show normal liver morphology though fed with high fat diet (60, 61). The current understanding of how osteocalcin regulates lipid accumulation is still unclear. One potential mechanism is through the receptor Gprc6a since *Gprc6a^-/-^* mice also develop hepatic steatosis (56).

*Ocn*-deficient (*Osc^-^/Osc^-^*) mice show reduced muscle mass (62). To further investigate the role of osteocalcin in muscle, the receptor Gprc6a was conditionally deleted in myofibers using Mck-Cre (63). Gprc6a may also serve as the osteocalcin receptor in skeletal muscle since *Gprc6a-*deficient mice have impaired exercise capacity and are resistant to osteocalcin treatment (56, 63, 64). The analysis showed that osteocalcin is necessary to increase exercise capacity. The level of circulating under-carboxylated osteocalcin increases during exercising. The increasing Ocn can increase nutrient uptake and ATP production. The potential mechanism of osteocalcin in regulating muscle function is by promoting the expression of interleukin-6 (IL-6) (65). Increased IL-6 level results in bone resorption and in turn, de-carboxylates osteocalcin (66). These studies outline a feed-forward loop between bone and muscle. Further, both Ocn levels and muscle mass decrease with aging. Treatment with osteocalcin augments exercise capacity in both young and old mice (63, 64). This suggests that osteocalcin is necessary and sufficient to maintain/increase muscle mass in older mice. However, Moriishi et al. recently showed that there are no differences in muscle weight or fiber area between *Ocn^-/-^* mice and wild-type littermates (30).

Another recent review also addressed some of the controversial actions of uncarboxylated osteocalcin in adipocytes and hepatocytes (26, 30). Although recombinant osteocalcin does show intriguing effects, another possibility is that osteocalcin-deficient mice might acquire compensatory mechanisms during development. For example, Gprc6a may use other ligands instead of osteocalcin. Similar to osteocalcin null mice, Gprc6a deficient mice also showed discrepancies in the metabolic functions as reported by different laboratories (30).

## Osteocalcin in Male Fertility

A distinct role of osteocalcin as an endocrine factor related to male fertility has been described. Oury and colleagues were the first to show that osteocalcin favors male fertility (41). Male *Osc^-^/Osc^-^* mice have lower litter frequencies, testis size and weights, and testosterone levels. These phenotypes caused by osteocalcin deficiency could be rescued by supplementing male mice with exogenous, uncarboxylated osteocalcin. Moreover, in the gain-of-function model of osteocalcin [*Esp^-/-^*, (40, 48, 67)], hyperandrogenism is noted. The study further identified the G-protein coupled receptor Gprc6a as the receptor for mediating testosterone synthesis. Mice lacking *Gprc6a* in Leydig cells showed decreased testis size, weight and testosterone levels. The role of osteocalcin in modulating male reproduction was also identified in humans by examining patients with primary testicular failure due to *GPRC6A* mutations (68). However, both Diegel et al. and Moriishi et al. showed discrepant results compared to Karsenty group regarding the role of osteocalcin in male fertility (*Bglap/2^dko/dko^* and *Ocn^-/-^*) (26, 30). Diegel and colleagues reported that there are no differences in testis size and blood testosterone levels between *Bglap/2^dko/dko^* and wild-type mice. Moriishi and colleagues also demonstrated that testis size, serum testosterone levels, testosterone synthesis and spermatogenesis are normal in *Ocn^-/-^* mice compared to wild-type mice. As discussed in detail above, side-by-side analysis of distinct strains of osteocalcin-deficient mice may be helpful to resolve the role of osteocalcin in male fertility in mice. In addition, careful review of potential methodologic differences between studies may help clarify these apparent discrepancies. Moreover, identification of additional humans with rare and common osteocalcin/GPRC6A variants linked to male fertility phenotypes is needed to further understand the role of osteocalcin as an LH-independent regulator of testicular testosterone synthesis. Additional (non-osteocalcin) ligands for Gprc6a have been proposed. As such, an improved structure/function understanding of how this receptor control Leydig cell testosterone synthesis, and how Gprc6a variants affect the function of this receptor (69), is needed (70).

## Osteocalcin in the Central and Peripheral Nervous System

Studies on a potential role for osteocalcin in the central nervous system (CNS) were prompted by the initial observation that *Osc^-^/Osc^-^* mice displayed an extremely passive phenotype during routine animal handling (27). Since this phenotype was present in both male and female mice, it was unlikely to be related to male-specific hypogonadism discussed above. Rather, it was noted that under-carboxylated osteocalcin could cross the blood-brain barrier where it accumulated in the midbrain and brainstem. Formal behavioral testing revealed anxiety-like phenotypes with learning defects in *Osc^-^/Osc^-^* mice (41). Neuroanatomic analysis of brains from *Osc^-^/Osc^-^* mice showed hippocampal atrophy (specifically in the dentate gyrus region) with frequent absence of the corpus callosum. These neuroanatomic abnormalities were associated with dramatic changes in overall CNS neurotransmitter levels: *Osc^-^/Osc^-^* mice show reduced brainstem monoamines and increased GABA levels. At the electrophysiologic level, *Osc^-^/Osc^-^* mice show increased activation potential of brainstem neurons in the locus coeruleus. Direct delivery of recombinant un-carboxylated osteocalcin into the CNS corrected molecular and phenotypic abnormalities in *Osc^-^/Osc^-^* mice. Interestingly, the role of osteocalcin in CNS development seems to be due to the ability of circulating maternal uncarboxylated osteocalcin to cross the placenta and the fetal blood-barin barrier. In humans, decreased circulating osteocalcin levels are associated with poor cognitive performance (71, 72). Highlighting the potential function of CNS-active un-carboxylated osteocalcin to restore cognitive function in aging, injection of plasma from young control, but not *Osc^-^/Osc^-^*, mice improved cognitive function and anxiety-related behaviors in aged mice. Osteocalcin appears to accomplish these effects by promoting synaptic transport of vesicles containing the neurotrophic factor BDNF in a pathway in hippocampal neurons involving the histone binding protein RbAp48 (73, 74).

While the peripheral extra-skeletal functions of osteocalcin appear to be mediated *via* GPRC6A, osteocalcin uses a distinct G protein coupled receptor to regulate neurons in the central and peripheral nervous system. GPR158 is a neuronally-expressed class C GPCR required for the CNS effects of osteocalcin. Mice lacking this GPCR in brain show similar learning defects and anxiety-related phenotypes as *Osc^-^/Osc^-^* mice, and studies using compound heterozygotes for both genes show that they function in a similar genetic pathway (73). In neurons, GPR158 signals through a Gαq-dependent pathway to promote IP_3_ generation. It remains possible that additional CNS osteocalcin receptors exist since the pattern of GPR158 expression does not perfectly match the pattern of CNS uncarboxylated osteocalcin binding.

In addition to its proposed role in the central nervous system, more recently it has also been reported that osteocalcin regulates output of parasympathetic neurons during the “fight or flight” (acute stress) response (ASR) (75). Exposure of rodents and humans to stressful stimuli acutely increases circulating bioactive osteocalcin levels in a manner dependent on the amygdala. Interestingly, treatment of osteoblasts with the excitatory neurotransmitter glutamate rapidly increases osteocalcin production, and glutamatergic nerve terminals can be found immediately adjacent to osteoblasts in bone. Therefore, the model emerges that the ASR increases sympathetic input to bone which in turn rapidly promotes osteoblastic osteocalcin production. In the ASR, *Osc^-^/Osc^-^* mice show blunted physiologic changes such as heat generation, increased oxygen consumption, and increased serum glucose levels. Osteocalcin promotes the acute stress response by binding to GPRC6A expressed in parasympathetic neurons. Upon binding to parasympathetic nerves, osteocalcin inhibits their firing and blunts parasympathetic tone and therefore promotes sympathetic output in the ASR. By linking bone and the acute stress response, osteocalcin may serve as an endocrine link between the ability of an organism to quickly run away from danger and promote physiologic changes to facilitate this rapid response (75).

Taken together, this line of investigation supports a role for bone-derived osteocalcin as a factor that modulates brain development and function, thereby linking bone homeostasis with whole body physiology and higher order cognitive functions (76). That being said, careful review of potential neuronal functions of osteocalcin in independent models, as reviewed above, will be helpful to build confidence in these findings. Future studies are needed to better define the role of the putative CNS osteocalcin receptor GPR158 in human nervous phenotypes, and to explore whether other CNS osteocalcin receptors may exist.

## Sclerostin

Sclerostin is a secreted glycoprotein expressed predominantly by mature osteocytes that is best known as a negative regulator of bone formation (77). Sclerostin was originally discovered because of inactivating mutations in the coding and enhancer regions of the *SOST* gene that cause the rare high bone mass disorders sclerosteosis and van Buchem disease (78). Although sclerostin was initially described as a BMP antagonist (79, 80), it is now primarily studied as a negative regulator of Wnt signaling (81). Sclerostin antagonizes Wnt signaling by occupying Wnt coreceptors LRP5/6 and preventing their binding to Wnt ligands, which inhibits downstream canonical Wnt signaling (78, 82). Sclerostin also binds directly to LRP4, a membrane-bound protein that facilitates its interactions with LRP5/6 (83). Since active canonical Wnt signaling promotes osteogenic differentiation and osteoblast maturation and survival, low sclerostin expression leads to bone anabolism whereas high expression inhibits bone formation (77). The regulation of sclerostin in bone in response to mechanical and biochemical cues and its role in regulating bone homeostasis has been reviewed elsewhere (79, 80).

The possibility that sclerostin can exert endocrine effects on non-skeletal tissues is inspired by the detection of sclerostin protein in circulation. Whereas sclerosteosis patients have no detectable protein in circulation, heterozygous carriers of *SOST* mutations have serum sclerostin levels approximately half that of control subjects (84) and bone mineral density (BMD) values higher than age-matched controls (85). In mice, sclerostin overexpression in the liver causes increases in serum sclerostin and loss of trabecular bone mass (86). Dozens of clinical studies have reported correlations between serum sclerostin levels and age, sex, bone mass, and disease (87). While most studies agree that circulating sclerostin increases with age (88–90), puzzling inconsistencies and contradictions exist, such as reports of both positive (89, 91, 92) and negative (93) associations between serum sclerostin and bone mass. The extent to which sclerostin measured in serum typically represents an active mediator of Wnt signaling versus a biomarker of ongoing disease (discussed more later), remains to be determined.

*Bona fide* endocrine effects of sclerostin would require that the protein be transported from the cell of origin to distant tissues. Sclerostin is a marker of mature osteocytes, and the osteocyte lacuno-canalicular network provides a pathway for secreted osteocyte factors to enter the circulation (94, 95). Inactivation of the *Sost* gene in mice with osteoblast and osteocyte-targeted Cre recombinases results in undetectable serum sclerostin, suggesting that osteocytes are the primary source of sclerostin in circulation (96). At least two clinical studies have found that circulating sclerostin does not correlate with *SOST* mRNA in bone biopsies (91, 97). However, the relationship between serum sclerostin and mRNA in bone biopsies is inherently sensitive to the mechanical environment of the biopsy site, the cortical/cancellous composition of the biopsy, and differences in clearance rates between subjects, so these studies do not necessarily detract from the animal work demonstrating that osteocytes are the primary source of circulating sclerostin. It is worth noting that *Sost* ablation with Prx1-Cre-mediated recombination does not fully reduce serum sclerostin, likely due to contributions from osteocytes in the axial skeleton. However, the high bone mass phenotype in the appendicular skeleton of these mice matches that seen with systemic *Sost* deletion better than Dmp1- and Col1-Cre models (96). Together these models indicate that sclerostin regulates bone mass through primarily paracrine signaling from osteoblasts, osteocytes, and additional cells derived from the limb mesenchyme. At various points in normal development and in disease, low levels of *SOST* mRNA or sclerostin protein have been detected in additional locations including cementocytes, hypertrophic chondrocytes, synovial fibroblasts, vascular smooth muscle cells, and kidney (79, 80, 98). There is no evidence to date to support that cells other than osteocytes contribute significantly to sclerostin in the circulation.

While questions remain regarding the accuracy and utility of measuring circulating sclerostin, many groups have gone on to test hypotheses regarding the role of sclerostin on non-bone tissues. Here we review the effects of sclerostin on adipose tissue, renal mineral metabolism, and the cardiovascular system. As more evidence is gathered to determine whether bone-derived sclerostin is implicated in these outcomes, the endocrine capacity of sclerostin will be decided.

## Sclerostin in Adipogenesis

Wnt signaling plays a key role in mesenchymal progenitor differentiation. Activation of Wnt signaling drives osteogenic differentiation of early mesenchymal precursors, while suppression of Wnt signaling promotes adipogenesis (99). Through its interactions with LRP4/5/6, sclerostin inhibits canonical Wnt signaling and could therefore stimulate adipogenesis. Studies of body adiposity in sclerosteosis patients have not been reported, and clinical descriptions that discuss patient weight compared to control subjects attribute slight increases to the extreme density of the skeleton (100). Nevertheless, correlations between circulating sclerostin and fat mass or metabolic disorders have inspired investigations into possible endocrine effects of sclerostin on adipose tissue.

### Clinical Evidence

Clinical studies demonstrate that serum sclerostin levels are positively correlated with fat mass and incidence of metabolic disorders. Serum sclerostin is positively correlated with abdominal fat mass, android and gynoid fat mass, and body mass index (BMI) in men and women (89, 92, 101) and with vertebral bone marrow fat content in men (102). Men and women with type 2 diabetes have significantly higher serum sclerostin than non-diabetic controls (103–105). Even in prediabetes, when insulin resistance and secretion are first altered, circulating sclerostin is elevated and positively correlated with fasting glucose production and metrics of insulin resistance (106). Inactivating mutations in *LRP6*, as well as single nucleotide polymorphisms (SNPs) in *LRP5* that are associated with low bone mass, are correlated with increases in metabolic syndrome and diabetes in humans, similar to mice that overexpress sclerostin (13, 107, 108). Individuals with a gain of function mutation in *LRP5* that causes high bone mass have decreased upper-to-lower body fat ratio and increased insulin sensitivity, consistent with the metabolic phenotype of mice that do not express *Sost* (13, 107). These studies support a role for sclerostin and Wnt signaling in the regulation of adipose tissue and whole-body metabolism, but they need to be interpreted with caution for several reasons.

First, cross-sectional/case-control studies are insufficient to demonstrate causal relationships between sclerostin levels and disease phenotypes. While individual-level data allows strong correlations to be drawn, these human studies lack interventions that would help to rule out confounding variables. None of the clinical trials evaluating therapeutic sclerostin-neutralizing antibodies (Scl Ab) to date report fat mass or insulin resistance as outcomes. This interventional design would be ideal for assessing the effects of sclerostin in humans. Second, the accuracy and consistency of assays for circulating sclerostin is not well established. Significantly different sclerostin concentrations have been found in serum and plasma from the same patients using three different assays (87). When two commercially available assays were compared directly, they also produced significantly different results (109). Care must therefore be taken when conducting meta-analyses of studies that utilize different sclerostin assays, and individual studies would ideally use a consistent assay for all subjects and report their validation strategy. Third, the extent to which circulating sclerostin levels represent active protein is not entirely certain. In cross-sectional studies, circulating sclerostin is often positively correlated with BMD even though high sclerostin levels would be expected to suppress bone formation (89, 91, 92). Mendelian randomization studies clarified this relationship, showing that high serum sclerostin is causally related to low BMD and that high BMD causes high serum sclerostin (93). In this case, serum sclerostin measurements may include both bioactive molecules and biomarkers of osteocyte activity. Furthermore, most studies do not investigate whether changes in serum sclerostin arise from changes in skeletal production or in renal clearance. Therefore, serum sclerostin levels and metabolic disorders may both reflect underlying differences in kidney function rather than pathogenic overproduction of sclerostin. Fourth, serum sclerostin measurements may be disrupted when Scl Ab is administered. Several animal studies that measure serum sclerostin after treatment with Scl Ab report increases in circulating sclerostin even though β-catenin activity and bone mass increase (13, 110). One potential explanation is that the antibodies used to quantify serum sclerostin detect the protein whether it is biologically active or bound and inactivated by the therapeutic neutralizing antibody (110). Well-controlled interventional studies will be needed in order to reliably correlate bioavailable sclerostin with the endocrine functions it exerts.

### *In Vitro* Evidence

*In vitro* experiments have demonstrated the direct effects of sclerostin on adipogenesis in primary and immortalized cells. Sclerostin positively regulates the differentiation of the 3T3-L1 preadipocyte cell line. Treatment with recombinant sclerostin or with osteocyte conditioned media increases expression of adipogenic transcription factors *Pparg* and *Cebpa* and increases lipid accumulation as measured by oil red O staining (111, 112). Primary adipocytes treated with recombinant sclerostin respond by increasing expression of adipocyte differentiation markers, increasing fatty acid synthesis, and increasing oil red O staining. Primary cells also reduce their metabolism of fatty acids in response to sclerostin, as evidenced by downregulation of genes such as *Cpt1a* and reduced oleate oxidation (13, 113). Mesenchymal stromal cells from mouse bone marrow, mouse ear tissue, and human bone marrow also respond to treatment with recombinant sclerostin and osteocyte conditioned media with increased expression of *Cebpa* and *Pparg* and increased oil red O staining (111). Thus when isolated *in vitro*, sclerostin consistently stimulates adipocyte differentiation and increases cell lipid content through enhanced fatty acid synthesis and reduced catabolism.

### Evidence From Animal Studies

The positive regulation of adipogenesis by sclerostin is further supported by studies of white adipose tissue and metabolism in animals. Mice with systemic ablation of *Sost* (*Sost^-/-^* mice) have lower overall fat mass as measured by qNMR and reduced mass of white adipose tissue from gonadal, inguinal, and retroperitoneal fat pads. Within the white adipose tissue of *Sost^-/-^* mice, individual adipocytes were also smaller, and Wnt target genes were upregulated (13). In mice injected with an AAV causing overexpression of sclerostin the same relationship was supported; Sost-AAV-treated mice had significantly increased fat mass, increased weight of individual fat pads, increased adipocyte size, and down-regulation of Wnt target gene expression in white adipose tissue depots (13).

The effects of sclerostin on white adipose tissue *in vivo* appear to be both pro-anabolic and anti-catabolic. Genes associated with adipocyte differentiation and lipid synthesis were increased in fat pads from mice treated with Sost-AAV and downregulated in *Sost^-/-^* mice (13). Fatty acid synthesis, as measured by ^3^H-acetate incorporation, was also reduced in *Sost^-/-^* mice. At the same time, genes associated with fatty acid oxidation and markers of beige adipocytes, such as *Cpt1a*, *Ppargc1a*, and *Ucp1*, were increased when Sost was ablated. Beige adipocytes are cells in white adipose tissue that express UCP1 and take on a thermogenic brown adipocyte-like phenotype with improved insulin sensitivity and glucose metabolism (114). *Sost^-/-^* mice and Scl Ab-treated mice (1 dose/week, 30 mg/kg, 8 weeks) show increased oxidation of the fatty acid oleate in adipose tissue explants (13). These findings are in agreement with *in vitro* studies on primary and immortalized adipocytes. However, they do not demonstrate whether sclerostin predominantly acts on adipocytes or on mesenchymal progenitor cells to induce adipogenesis.

The ability of sclerostin to act directly on adipose tissue *in vivo* was investigated in mice made conditionally insensitive to sclerostin with deletion of *Lrp4* from white and brown adipocytes (83). These mice showed small adipocytes with reduced expression of *Cebpa*, though fat mass and weight of individual fat pads was not affected. By further reducing sclerostin availability with compound heterozygous mice expressing one allele of *Lrp4* in adipocytes and one allele of *Sost* systemically, fat mass and adipocyte size were reduced (113). Two studies found that deletion of *Lrp4* from osteoblast-lineage cells leads to high bone mass and increased circulating sclerostin, due to either increased sclerostin production in bone or the loss of local sequestration by LRP4 (113, 115). This model of high circulating sclerostin also increased fat mass in white adipose tissue depots (113). These studies indicate that sclerostin can act directly on adipocytes *in vivo*, but progenitor cells targeted by circulating sclerostin during development likely also contribute to the full white adipose tissue phenotype of mice with systemic *Sost* overexpression or ablation.

Sclerostin may also play a broader role in metabolism in mice. Feeding wild-type mice with a high fat diet for 4 or 8 weeks leads to increased white adipose tissue mass, increased *Sost* mRNA in bone, and increased serum sclerostin (13). Leptin-deficient *ob/ob* and *db/db* mice also have elevated serum sclerostin compared to wild-type littermates (13). *Sost^-/-^* mice fed a high fat diet, however, gain less body weight and fat mass than their wild-type counterparts. Scl Ab treatment (1 dose/week, 30 mg/kg, 8 weeks) also partially protected mice from high fat diet-induced increases in body weight and fat mass. Along with differences in white adipose tissue mass, *Sost^-/-^* mice showed improved glucose handling in glucose tolerance and insulin sensitivity tests compared to wild-type littermates on normal or high fat diets. These results are supported in Scl Ab-treated mice. Sost-AAV-treated mice, on the other hand, have higher random-fed insulin levels, are worse at regulating blood glucose levels, and are less sensitive to insulin (13). Reducing adipocyte sensitivity to sclerostin through *Lrp4* deletion and additionally reducing circulating sclerostin both cause improvements in glucose handling, suggesting that sclerostin’s effects on adipose tissue can have a systemic impact (113).

In addition to these endocrine effects on white adipose tissue, sclerostin also positively regulates adipose tissue in the bone marrow. Marrow adipose tissue - distinct from the white adipose tissue found in subcutaneous and visceral fat depots - is dynamically regulated with age, diet, hormones, and disease, though its function in skeletal homeostasis is still being determined (114). Effects of sclerostin on immune cells in the marrow, recently reviewed elsewhere (116), and on marrow adipose tissue may be best classified as paracrine, but we will discuss the available research on marrow adipose tissue here.

Consistent with the effects of *Sost* ablation on white adipose tissue, *Sost^-/-^* mice have decreased bone marrow adipose tissue volume as detected by osmium tetroxide micro-CT (117). Likewise, mice treated with Scl Ab for 3 weeks (1 dose/week, 100 mg/kg) end with lower marrow adipocyte number and size (118). In a separate study, 3 weeks of Scl Ab treatment (2 doses/week, 25 mg/kg) did not significantly affect bone marrow adipocyte number in healthy mice, nor did it decrease the number of adipocytes surrounding a fracture callus (110). These studies all used young male mice (6-11 weeks old), though the differences in animal age and antibody dose may explain the discrepancy in outcomes.

Male and female rats treated with Scl Ab (1 dose/week, 3 or 50 mg/kg) for 4 or 26 weeks presented a more complex response (119). After 26 weeks of treatment, both sexes showed significant dose-dependent decreases in tissue adiposity driven by decreased adipocyte number, consistent with prior studies in mice. However, when adipocyte numbers were normalized to bone marrow area, which decreases as bone mass increases in treated animals, the significant effects of sclerostin antibody on marrow adiposity were limited to males. Whether normalized to tissue area or marrow area, marrow adiposity of female rats increased in the first four weeks of Scl Ab treatment, again in a dose-dependent manner, whereas male rats showed no changes in the first four weeks of treatment. Sex-dependent differences in baseline trabecular bone volume and marrow adiposity may drive the sex-specific differences in early response to Scl Ab. Also notable was that marrow adiposity increased with age, an effect that was only partially blocked by Scl Ab treatment. In sum, these studies illustrate a predominantly positive relationship between sclerostin and marrow adiposity, but reconciling the age-, sex-, and dose-dependent effects of Scl Ab treatment will require further study.

In disease models where marrow adiposity is elevated, a protective role of sclerostin antibody treatment has been reported. Rabbits treated with Scl Ab for five months (2 doses/week, 13 mg/kg) were protected from the increase in marrow fat fraction caused by ovariectomy. While adipocytes in untreated ovariectomized rabbits increased in diameter and density, those in rabbits that also received Scl Ab were indistinguishable from sham-operated animals (120). Streptozotocin-induced diabetic mice have higher bone marrow adiposity than non-diabetic mice. In this model, Scl Ab treatment slightly attenuates the additional increase in adipocyte density seen around a fracture callus (3 weeks, 2 doses/week, 25 mg/kg) (110). This raises the possibility that sclerostin acts as a mediator in disease even if it is not a primary regulator of adipogenesis in bone marrow. Additional *in vivo* studies with conditional deletion of LRPs from adipocytes and from mesenchymal progenitor cells are needed to determine the extent to which sclerostin acts directly on marrow adipocytes versus altering the commitment of bipotential precursor cells to cause these phenotypes.

*In vitro* studies, animal experiments, and clinical trials have provided evidence that sclerostin can act on adipose tissue to regulate Wnt signaling, adipogenesis, and metabolism. Together this body of work suggests an overall positive regulatory relationship between sclerostin and adipogenesis, but the extent of its regulatory capabilities and its importance relative to other endocrine and paracrine factors in homeostasis and in disease is not entirely clear. In humans, all studies published to date linking sclerostin levels and metabolic phenotypes are associative. While *in vitro* and *in vivo* studies show that sclerostin is capable of regulating adipocyte behavior, its importance within physiological concentrations and in concert with other signaling molecules is not yet clear. In mouse models, global deletion of sclerostin or mutations in Wnt signaling-related proteins may cause secondary changes in bone that in turn regulate adipocyte biology, rather than direct effects of sclerostin protein on adipocytes. The careful experiments describing cell type-specific contributions of *Sost* to regulation of bone mass (96) have not been conducted with adipose outcomes. Future studies using bone-specific sclerostin mutant animals will be helpful to isolate the effects of bone-derived sclerostin on distant tissues in mice.

## Sclerostin in Mineral Metabolism

The extreme high bone mass phenotype seen in people with sclerosteosis and in *Sost^-/-^* mice likely requires a shift in systemic mineral homeostasis to support absorption and retention of the required calcium and phosphorus. In neutral calcium balance, the dietary calcium absorbed in the intestines is offset by the amount of calcium filtered and then reabsorbed or excreted *via* the kidneys. Calcium and phosphorus balance are carefully maintained through the coordinated control of circulating hormones including PTH, FGF-23, and vitamin D (121, 122). Since sclerostin plays a major role in regulating bone homeostasis, whether this same factor may modulate renal mineral metabolism or the actions of calciotropic hormones is of particular interest.

Indeed, *Sost^-/-^* mice demonstrate alterations in mineral metabolism resulting in enhanced absorption and reduced excretion of calcium and phosphorus. Active vitamin D (1α,25(OH)_2_D) concentrations were significantly elevated in the serum of *Sost^-/-^* mice, which would be expected to promote calcium and phosphorus absorption in the kidney and intestines. Accordingly, urinary calcium excretion was reduced, serum phosphorus was elevated, and serum FGF-23, which promotes phosphate excretion, was low in *Sost^-/-^* mice (14). In wild-type mice and in a mouse model of X-linked hypophosphatemia, sclerostin-neutralizing antibody treatment (4 weeks, 2 doses/week, 25 mg/kg) significantly increased serum phosphate and decreased circulating FGF-23 (123). Therefore, sclerostin deficiency leads to alterations in mineral metabolism to enhance absorption and reduce excretion of calcium and phosphorus.

**Figure 2:**
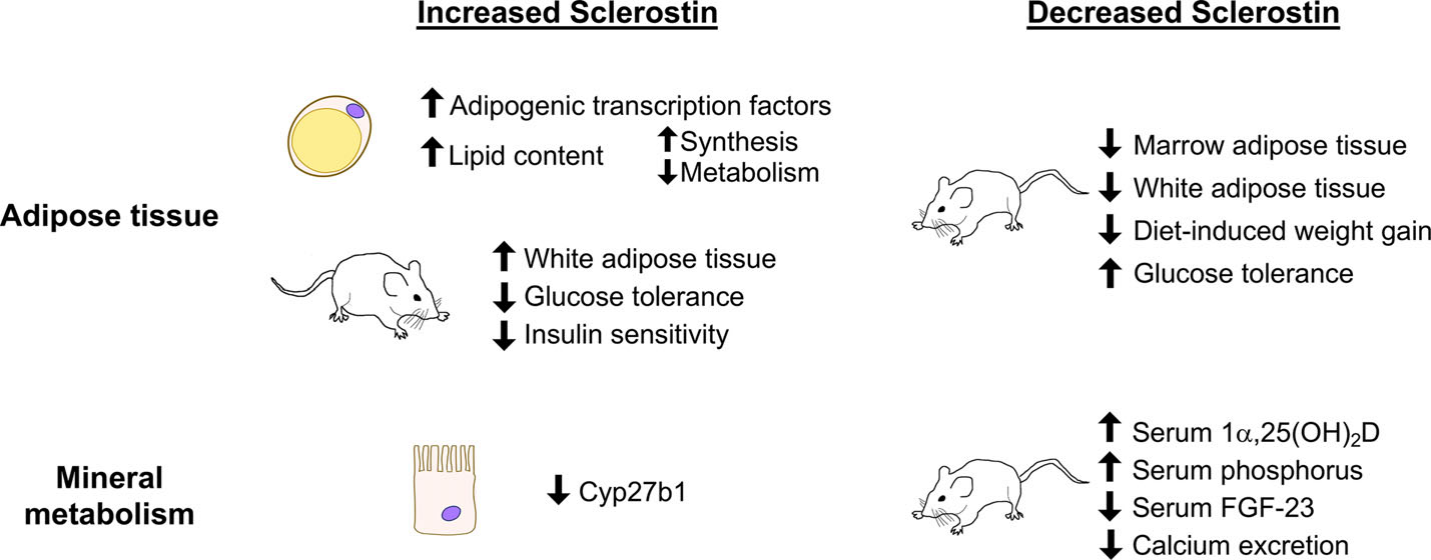
Summary of sclerostin-mediated effects on adipose tissue and on mineral metabolism. Recombinant sclerostin exerts direct effects on adipocytes and kidney proximal tubule cells in culture. Mice overproducing sclerostin demonstrate increased adiposity and reduced glucose tolerance. Reduced sclerostin *in vivo*, achieved through inactivating mutations, conditional ablation, or neutralizing antibodies, leads to reduced adiposity, improved glucose handling, and altered mineral metabolism in mice.

Since vitamin D regulates mineral absorption in both the kidney and the intestines, the possibility of direct action of sclerostin on vitamin D metabolism has been investigated. Inactive vitamin D prehormone (25(OH)D) must be converted to the active form (1α,25(OH)_2_D) by 25-hydroxyvitamin D 1α-hydroxylase (1α-hydroxylase, encoded by *Cyp27b1*), which primarily occurs in the kidney. *Cyp27b1* expression is slightly elevated in the kidneys of *Sost^-/-^* mice and is significantly repressed in proximal tubule cells after treatment with recombinant sclerostin, consistent with the elevated levels of 1α,25(OH)_2_D in serum of *Sost^-/-^* mice (14). High serum PTH, low serum phosphorus, and low FGF-23 could also cause high 1α-hydroxylase activity, but of these factors, *Sost^-/-^* mice only have low serum FGF-23 (124–126). Together these results suggest that sclerostin, through direct effects on proximal tubule cells and indirectly through FGF-23, negatively regulates the synthesis of 1α,25(OH)_2_D. The ability of sclerostin to regulate systemic mineral metabolism may therefore be regulated by a combination of vitamin D-mediated effects and direct action on the kidney to control calcium excretion. The potential endocrine effects of sclerostin on mineral metabolism and on adipogenesis as studied in cell and animal models are shown in **Figure 2**. For schematics describing the role of sclerostin in bone, we again refer the reader to existing reviews (79, 80).

To date, the effects of sclerostin overexpression on mineral metabolism remain to be determined, and many outstanding questions remain regarding potential renal actions of sclerostin (127). For example, does FGF-23 act independently of sclerostin or mediate its effects on 1α,25(OH)_2_D synthesis? Which nephron segment in the kidney responds to sclerostin to regulate calcium excretion? Do these processes involve canonical Wnt signaling? The extent to which sclerostin functions as a normal part of processing dietary calcium, versus only in a disease state, also remains to be determined. Supporting the importance of sclerostin in the kidney, though, a meta-analysis of genomewide association studies found robust association between SNPs in *B4GALNT3*, which is highly expressed in the kidney, and serum sclerostin (93). Thus, the kidney may participate in regulating the levels of circulating sclerostin available to act on it by modulating expression of *B4GALNT3*. Further mechanistic studies are needed to validate the role of renal *B4GALNT3* in regulating sclerostin availability and clearance.

Studies with adult sclerosteosis patients have found normal urinary calcium excretion, plasma PTH, 25(OH)D prehormone, serum calcium, and serum phosphorus (84, 128). van Lierop et al. reviewed 96 cases of sclerosteosis and determined that active 1α,25(OH)_2_D is elevated in sclerosteosis patients compared to carriers (129). Children with sclerosteosis have high serum phosphorus and calcium, and they may better match the developmental stage of mice used in research (128). Interpretation of clinical trials studying sclerostin-neutralizing antibodies blosozumab and romosozumab is complicated by the simultaneous administration of calcium and vitamin D supplements. Blosozumab caused slight elevation of serum 1α,25(OH)_2_D and PTH compared to placebo that did not reach statistical significance (130). Romosozumab caused a transient decrease in serum calcium and a slight dose-dependent increase in serum PTH compared to baseline, but vitamin D was not reported after baseline (131, 132). In the first single-dose studies of romosozumab (then called AMG 785) when calcium and vitamin D supplements were not given, serum calcium and PTH reacted similarly, but vitamin D was not measured (133). In the relatively healthy people studied in these trials, sclerostin deficiency appears to have a small effect on mineral metabolism. However, given the careful balance of sclerostin, PTH, and FGF-23 [reviewed by (134)], sclerostin may play a bigger role in patients with chronic kidney disease (135).

Because of the frequency of bone phenotypes in patients with chronic kidney disease, the role of circulating sclerostin has been studied extensively in this population. In two cross-sectional studies of patients with chronic kidney disease, serum sclerostin levels were higher in patients with the lower glomerular filtration rates characteristic of advanced disease (136, 137). Rather than reflecting reduced renal clearance, however, the urinary excretion of sclerostin also increased as kidney disease progressed (137). This along with immunohistochemistry of bone biopsies from chronic kidney disease patients suggests that increased production of sclerostin by osteocytes may be a factor in the development of chronic kidney disease (138, 139). This is further supported by bone biopsies from patients undergoing kidney transplant. Serum sclerostin measurements were significantly correlated with the percentage of sclerostin-positive osteocytes measured by immunohistochemistry and negatively associated with residual renal function in end-stage kidney disease (140). As needed with studies of adiposity, careful interventional clinical trials and animal studies with tissue-specific sclerostin overexpression or ablation will be necessary to isolate the effects of sclerostin on mineral metabolism.

## Sclerostin in Vascular Calcification

The individual effects of sclerostin on bone, adipose tissue, and kidney suggest that sclerostin-neutralizing antibodies should have net positive benefits on human health, and clinical trials thus far have reported few adverse events from sclerostin-neutralizing antibodies (130–132, 141, 142). While sclerosteosis can lead to life-threatening elevation of intracranial pressure caused by thickening of the skull, health of people with sclerosteosis and van Buchem’s disease is considered good overall, without involvement of the heart or lungs (143). Furthermore, sclerosteosis carriers have high bone mass without intracranial hypertension. However, a numeric imbalance in serious cardiovascular adverse events was reported in romosozumab-treated women compared to alendronate-treated women in the ARCH trial (144) as well as in romosozumab-treated men compared to placebo-treated men in the BRIDGE trial (145). While the absolute risk for adverse cardiovascular events remained quite small, these findings led the U.S. FDA to include a black box packaging warning for potential cardiac risks associated with romosozumab. It is not yet known why this effect on cardiovascular adverse events was not apparent in women in the placebo-controlled trial (131). It is possible that the placebo-controlled trial in women included younger patients with fewer risk factors for cardiac events or that alendronate is somewhat cardioprotective. These clinical findings also raise the possibility that circulating sclerostin exerts effects on the vasculature in a cardioprotective manner.

A recent genetics study supports the idea that inhibition of sclerostin elevates risk of cardiovascular adverse events (15). Bovijn et al. found that two common *SOST* variants associated with high BMD were also associated with reduced expression of *SOST* and a small increase in lifetime risk of myocardial infarction, coronary heart disease, and other adverse cardiovascular events. One mechanism by which sclerostin could prevent cardiovascular adverse events is through inhibition of vascular calcification, though correlations between sclerostin and vascular calcification have identified both positive and negative relationships (146). In support of a protective role for sclerostin, a multivariate logistic regression model of patients with chronic kidney disease found that lower circulating sclerostin was significantly associated with aortic calcification (147). In another human study, biopsies from aortic aneurysms contained less sclerostin protein than healthy aortic tissue (148). *ApoE^-/-^* mice stimulated with angiotensin II develop aortic aneurysms and atherosclerotic plaques, but when these mice also overexpressed human sclerostin or were injected with recombinant mouse sclerostin they were protected from aortic aneurysms and atherosclerosis (148).

On the other hand, sclerostin is also positively associated with cardiovascular disease in some cases. When vascular smooth muscle cells are induced toward calcification *in vitro*, they express more sclerostin (149). In a mouse model of chronic kidney disease-mineral and bone disorder, aortic calcification and circulating sclerostin were significantly increased compared to healthy mice (150). In a mixed population of people with and without type 2 diabetes, serum sclerostin was positively associated with the presence of aortic calcifications and cardiovascular mortality over the eight-year longitudinal study (151). Finally, in end-stage renal disease, coronary artery & epigastric artery calcification positively correlated with serum sclerostin. Since no specific sclerostin mRNA or protein expression was detected in the vessels, it appeared that circulating sclerostin produced elsewhere was responsible for the vascular calcification (152). In these associative studies, it is still not known whether sclerostin is involved in the cause or response to calcification. Furthermore, many of these studies were performed in the context of chronic kidney disease where sclerostin also correlates with age, male gender, and glomerular filtration rate, so its individual effect on the vasculature is difficult to discern.

A safety study sponsored by Amgen Inc., Astellas, and UCB Pharma reported no effects of sclerostin-neutralizing antibody on the cardiovascular system in multiple animal models (153). Healthy rats and cynomolgus monkeys as well as angiotensin II-infused *ApoE^-/-^* mice and ovariectomized *ApoE^-/-^* mice on a high fat diet were administered romosozumab and monitored for toxicity and cardiovascular function. In healthy animals and in mouse models of atherosclerosis, effects of sclerostin neutralization on cardiovascular function, vascular calcification, and transcription were nonsignificant. Only the supraphysiological dose of 300 mg/kg romosozumab in monkeys elicited a response - sporadic increases in heart rate and blood pressure – which was discounted by the authors. While this study gives ample evidence that romosozumab is unlikely to exert direct effects on the cardiovascular system, many questions remain regarding the extent to which sclerostin itself functions in the vasculature and the mechanisms by which romosozumab induces cardiovascular adverse events in humans. The effects of sclerostin on cardiovascular health will doubtless be a focus of research in the future as more patients are prescribed sclerostin-neutralizing antibodies to increase bone mass and reduce fracture risk.

Further work will be needed to determine the extent to which bone-derived sclerostin regulates adipogenesis, renal mineral metabolism, cardiovascular health, and other potential non-skeletal tissues. Of particular importance are animal studies in which sclerostin is ablated specifically from osteoblast lineage cells, animal studies in which potential target cells are rendered insensitive to sclerostin, and clinical trials that measure the effects of sclerostin or sclerostin neutralizing antibodies on non-skeletal tissues in a prospective, interventional manner. As the full endocrine capacity of sclerostin is elucidated, it may lead to exciting new roles for osteocytes in systemic homeostasis.

## Conclusions and Future Perspectives

As summarized here, multiple extra-skeletal functions of osteocalcin and sclerostin have been proposed based on preclinical models. Given the central role of bone in whole organism physiology, it is not surprising that crosstalk between the skeleton and other organs exists and is mediated by bone-specific factors. It is likely that osteocalcin and sclerostin represent the tip of the iceberg with respect to how bone-derived factors regulate the function of other organs. As outlined above, future studies are needed to validate findings from preclinical models and to rigorously test hypotheses in humans. In addition, it will be important to consider how the organs targeted by bone-derived factors signal to the skeleton in order to maintain organismal homeostasis.

## Author Contributions

JW, CM, and MW wrote and edited the manuscript. All authors contributed to the article and approved the submitted version.

## Funding

CM acknowledges support from the NIH (T32DK007028). MW acknowledges support from the American Society of Bone and Mineral Research (Rising Star award) and the NIH (DK116716 and DK011794).

## Conflict of Interest

MW receives research funding from Radius Health and Galapagos NV on projects unrelated to this review. These funders had no role in preparation of this review manuscript or decision to publish.

The remaining authors declare that the research was conducted in the absence of any commercial or financial relationships that could be construed as a potential conflict of interest.
